# Local replication of simian immunodeficiency virus in the breast milk compartment of chronically-infected, lactating rhesus monkeys

**DOI:** 10.1186/1742-4690-7-7

**Published:** 2010-02-01

**Authors:** Sallie R Permar, Helen H Kang, Andrew B Wilks, Linh V Mach, Angela Carville, Keith G Mansfield, Gerald H Learn, Beatrice H Hahn, Norman L Letvin

**Affiliations:** 1 Division of Viral Pathogenesis, Beth Israel Deaconess Medical Center, Harvard Medical School, Boston, Massachusetts 02215, USA; 2 Children's Hospital Boston, Harvard Medical School, Boston, Massachusetts 02115, USA; 3 New England Regional Primate Center, Harvard Medical School, Southborough, Massachusetts 01772, USA; 4 Department of Medicine, University of Alabama at Birmingham, Birmingham, Alabama 35294, USA

## Abstract

Breast milk transmission remains a major mode of infant HIV acquisition, yet anatomic and immunologic forces shaping virus quasispecies in milk are not well characterized. In this study, phylogenic analysis of envelope sequences of milk SIV variants revealed groups of nearly identical viruses, indicating local virus production. However, comparison of the patterns and rates of CTL escape of blood and milk virus demonstrated only subtle differences between the compartments. These findings suggest that a substantial fraction of milk viruses are produced by locally-infected cells, but are shaped by cellular immune pressures similar to that in the blood.

## Findings

While transmission via breastfeeding remains a significant mode of infant HIV acquisition, the mechanisms of this transmission are not well understood. The level of both cell-free and cell-associated virus in milk have been linked to the risk of HIV infection of breastfeeding infants [1-3]. Therefore, viral variants in the milk are a likely source of transmitted virus.

Recent studies suggest that mucosal compartments are distinct from systemic compartments in their HIV/SIV-specific immune responses and virus quasispecies [4-8]. The immune response in milk may shape compartment-specific virus, as seen in other anatomic compartments such as semen and cervicovaginal fluid [6,9-11]. One study of the genetic diversity of milk virus suggested a difference in the dominant virus species in milk and peripheral blood [4]. A second study subsequently reported that the predominant virus in blood and milk were genetically similar, suggesting an equilibrium of virus between these compartments [12]. Thus, the production site of the virus transmitted via breastfeeding and the selection pressures that shape its genetic composition are not well understood.

It is well established that HIV/SIV-specific cellular immune responses are critical for control of systemic virus replication [13] and progression to AIDS [14]. While HIV/SIV-specific cellular immune responses have been identified in milk [15,16], the role of these responses in containing local virus replication is unknown. Immunodominant cytotoxic T lymphocyte (CTL) epitopes of SIV and HIV are under significant cellular immune pressure, leading to distinct mutations within these epitopes that facilitate virus escape from CTL recognition. Therefore, the rate at which these CTL escape mutations occur within a virus population may be indicative of the magnitude of the cellular immune pressure in that compartment. However, rates of CTL escape of mucosal and systemic virus quasispecies have not previously been compared. As the proportion of SIV-specific CD8+ T lymphocytes is two to three times higher in milk than in blood during acute SIV infection [15], a faster rate of CTL escape of the milk virus quasispecies than that in blood would indicate that this immune response in milk is acting on locally replicating virus.

The SIV-infected rhesus monkey model of HIV pathogenesis is an excellent model in which to study virus-specific immune responses and virus evolution in mucosal compartments, including breast milk [15]. In the present study, we sought to evaluate genetic similarities between virus quasispecies in blood and milk of chronically SIVmac251-infected rhesus monkeys through phylogenetic comparison of SIV envelope (*env*) sequences. We then compared the rate of CTL escape of two Mamu-A*01-restricted immunodominant epitopes (Tat TL8 and Gag p11C) in blood and milk virus. Animals were maintained in accordance with the guidelines of the "Guide for the Care and Use of Laboratory Animals" (National Research Council, National Academic Press, Washington, D.C., 1996).

### Groups of identical or nearly identical viruses in breast milk of chronically SIV-infected, lactating rhesus monkeys

**Figure 1:**
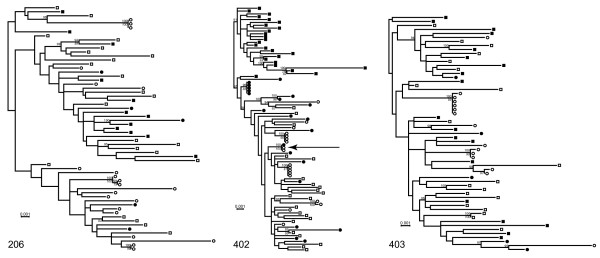
**Identification of groups of identical or nearly identical envelope sequences in breast milk of lactating, chronically SIV-infected rhesus monkeys**. Trees were inferred by maximum likelihood methods [19]. Cell-associated and cell-free blood virus quasispecies *env *sequences are indicated with closed and open squares, respectively, and cell-associated and cell-free milk virus quasispecies *env *sequences are indicated with closed and open circles, respectively. Bayesian posterior probabilities are shown above the branch and bootstrap support values are shown in italics below the branch. Sequences are shown for three chronically infected rhesus macaques: 206 (week 74 after SIV infection), 402 (week 67 after SIV infection), and 403 (week 49 after SIV infection).

Cell-free and cell-associated SIV *env *amplicons were sequenced from plasma, peripheral blood mononuclear cells (PBMC), breast milk supernatant, and breast milk cells obtained from 3 chronically SIV-infected, lactating rhesus monkeys within a 7 day period between 1 and 1.5 years after SIVmac251 infection. SIV *env *cassettes containing the *env *open reading frame were amplified by single genome amplification (SGA) to reduce the possibility of PCR-induced recombination during amplification [17]. A nested PCR was performed on end-point diluted virus cDNA, extracted as previously described [15], with primers: outer forward (5' - GAAAGGCTGTAGATGTCTAGG - 3'), outer reverse (5' - CTCATCTGATACATTTACGGGG - 3'), inner forward (5' - GGGTAGTGGAGGTTCTGGAAG - 3'), inner reverse (5' - CCCTACCAAGTCATCATCTTC - 3') (GenBank D01065). Total SIV copy numbers measured by quantitative RT-PCR [15] ranged from 6.9 × 10^3 ^to 1.5 × 10^6 ^copies/ml (3.3 × 10^3 ^to 2.2 × 10^5 ^total copies measured) in breast milk and from 5.25 × 10^5 ^to 2.1 × 10^7 ^copies/ml (4.7 × 10^4 ^to 1.8 × 10^6 ^total copies measured) in plasma. These total SIV copy numbers were measured in the animals at the time of collection of the samples used for sequencing. Sequences were trimmed to the start codon of ENV and to the 3' region of *env *with unambiguous sequencing for each amplicon within the same monkey (length: 2397-2559 base pairs) and aligned using ClustalW version 2 [18]. PhyML version 3.0.1 [19] was used to infer the evolutionary model parameters and phylogenetic trees. For each analysis, a GTR+I+G model (general time reversible model with invariant sites and gamma-distributed site-to-site rate variation) was used. Bootstrap support was based on 100 resamplings. Bayesian posterior probabilities were calculated using MrBayes version 3.1.2 [20] with chains of 2.1 to 3 × 10^7 ^iterations and 25% burn in (Fig. 1).

Overall, the range of nucleotide sequence diversity of cell-free milk virus (monkey 206 = 1.03%, monkey 257 = 0.76%, monkey 403 = 0.75%) was similar to that in the cell-free blood virus (monkey 206 = 1.1%, monkey 257 = 0.85%, monkey 403 = 0.73%). Strikingly, groups of identical or nearly identical cell-free *env*s were identified in milk of all animals, while this was not observed for plasma-derived cell-free *env *sequences. These groups of *env*s were either identical or differed by a single nucleotide (0 - 0.04% nucleotide difference) and comprised one-third to three-quarters of the cell-free milk virus amplified in each animal (monkey 206 = 38.1%, monkey 257 = 76.5%, monkey 402 = 73.3%). Moreover, in animal 402, a single breast milk cell-associated virus variant had a sequence identical to that of a cluster of cell-free milk viruses, which suggests that the clonal amplification of cell-free milk virus variants is produced by a small number of productively-infected, resident cells. In fact, our findings are consistent with a recent report that actively replicating virus in breast milk evolves under drug pressure that is distinct from that in the plasma [21]. Thus, locally-produced virus is likely the major source of virus present in breast milk.

However, we did not observe phylogenetic evidence of compartmentalization of blood and milk virus quasispecies, as the blood and milk viral variants were interspersed in the phylogenetic tree of sequences derived from each monkey. While the milk viruses did not cluster in the phylogenetic trees, all animals exhibited significant compartmentalization between blood and milk cell-free virus quasispecies as determined by the Slatkin-Maddison compartmentalization test of the minimum possible number of inter-compartment migration events compared to the distribution of migration events in 1000 randomized trees [22,23] implemented by HYPHY software [24] (monkey 206: p = 0.001; monkey 402: p = 0.003; monkey 403: p = 0.001). When repeated identical and nearly identical sequences were removed from the data set subjected to the Slatkin-Maddison analysis, significant compartmentalization between blood and breast milk was only evident in animal 206 (monkey 206: p = 0.006; monkey 402: p = 0.795; monkey 403: p = 0.505). Therefore, whether breast milk and blood represent distinct anatomic compartments for SIV in the setting of chronic infection remains an open question.

### Rate of CTL escape at immunodominant epitopes Tat TL8 and Gag p11C in breast milk virus

As a robust SIV-specific CD8+ T lymphocyte response occurs in breast milk during acute SIV infection, we investigated whether CTL escape of two Mamu-A*01-restricted immunodominant epitopes Tat TL8 and Gag p11C occurred at a different rate in milk and blood virus quasispecies. The Tat TL8 epitope was amplified by SGA with primers described above (6 to 31 amplicons per compartment). The Gag p11C epitope was analyzed by SGA with primers: outer forward (5' - GTCTGCGTCATCTGGTGCATTC - 3'), outer reverse (5' - TGTTTGTTCTGCTCTTAAGCTTTTGTAG - 3'), inner forward (5' - CAAAACAGATAGTGCAGAGACACCTAGTG - 3'), inner reverse (5' - GAAATGGCTCTTTTGGTCCTT - 3'). Virus quasispecies with at least one nonsynonymous mutation within an immunodominant epitope was defined as a CTL escape variant. CTL escape at the Tat TL8 epitope occurs rapidly in the blood so that all virus quasispecies have a mutated Tat TL8 epitope by day 28 of infection [25], whereas CTL escape at the Gag p11C epitope occurs much later due to structural constraints on the virus at that epitope [26,27].

CTL escape occurred in milk virus at both the Tat TL8 and Gag p11C epitope with patterns of epitope position mutations similar to those that occurred in the blood virus (data not shown). Although there were subtle differences in the rate of CTL escape of the milk and blood virus populations, these did not achieve statistical significance. At day 18 after infection, a lower proportion of virus remained wild type at the Tat TL8 epitope in blood (median = 34.3%; range = 12.5% - 90.9%) than in breast milk (median = 67.6%, range = 31% - 100%) of all monkeys (Table 1). However, CTL escape at the Tat TL8 epitope occurred in all virus variants from both compartments by day 28 after infection in all monkeys.

**Table 1:** CTL escape of breast milk and blood virus quasispecies at the Tat TL8 epitope during acute SIV infection.

		Days after SIV infection			
**Animal #**		**Day 14**	**Day 18**	**Day 21**	**Day 28**

**206**	blood	100%^a ^(17/17)^b^	14.3% (3/21)	0% (0/26)	ND^c^
	milk	100% (30/30)	87.5% (28/32)	17.4% (4/23)	0% (0/17)

**257**	blood	100% (29/29)	90.9% (20/22)	38.5% (10/26)	0% (0/20)
	milk	100% (21/21)	100% (30/30)	54.5% (12/22)	0% (0/31)

**402**	blood	100% (28/28)	12.5% (3/24)	7.7% (2/26)	ND
	milk	100% (18/18)	31.0% (9/29)	16.7% (1/6)	0% (0/32)

**403**	blood	100% (29/29)	54.2% (13/24)	9.4% (3/32)	ND
	milk	100% (23/23)	47.6% (10/21)	17.4% (4/23)	0% (0/31)

^a ^% reported is the proportion of virus quasispecies that did not have an amino acid substitution within the Tat TL8 epitope (remained wild type).

^b ^In parentheses is the number of virus quasispecies that remained wild type at the Tat TL8 epitope/number of virus quasispecies sequenced.

^c ^ND = not done

We used the Spearman's rank correlation test to determine if the proportion of virus variants with CTL escape of Tat TL8 at day 18 after SIV infection was associated with the proportion of Tat TL8-specific CD8+ T lymphocytes or the magnitude of the milk virus load [15]. The proportion of virus with evidence of CTL escape in the milk virus population most strongly correlated with the peak milk virus load (R^2 ^= 0.986, p = 0.083). Therefore, the slightly slower rate of CTL escape mutation of the Tat TL8 epitope in milk is likely a result of a lower rate of virus replication in the breast milk compartment.

As predicted by previous studies of CTL escape at the Gag p11C epitope, CTL escape at the Gag p11C epitope occurred at amino acid position 2 during chronic infection in both blood and milk virus populations (between weeks 17 and 46 after SIV infection) [27]. Interestingly, CTL escape appears to have occurred more quickly at Gag p11C in milk than in blood of 2 of 4 monkeys included in this study. The majority of the milk virus quasispecies was mutated at Gag p11C by week 37 in monkey 206 and week 41 in monkey 257, whereas the majority of blood virus quasispecies remained wild type at Gag p11C until week 45 and 46, respectively (Table 2) We were unable to detect more rapid CTL escape at the Gag p11C epitope in the milk virus population in one rapid progressor monkey who died less than one year after SIV inoculation (animal 403) and one monkey that was not lactating at the time of Gag p11C CTL escape (animal 402). In addition, there was no detectable correlation between the proportion Gag p11C-specific CD8+ T lymphocytes or milk virus load and the rate of Gag p11C CTL escape in milk using nonparametric linear regression analysis.

**Table 2:** CTL escape at the Gag p11c epitope in breast milk and blood virus quasispecies during acute SIV infection.

		Weeks after SIV infection						
Animal #		**Week 33**	**Week 35**	**Week 36**	**Week 37**	**Week 38**	**Week 40**	**Week 45**

**206**	blood	100%^a^ (14/14)^b^	65.4% (17/26)	59.4% (19/32)	61.5% (16/26)	41.7% (10/24)	30.4% (7/23)	8.3% (2/24)
	milk	80% (4/5)	29.0% (9/31)	83.3% (35/42)	3.1% (1/32)	ND^c^	ND	ND

		**Week 37**	**Week 39**	**Week 41**	**Week 42**	**Week 45**	**Week 46**	

**257**	blood	100% (16/16)	ND	ND	100% (28/28)	21.2% (7/33)	0% (0/17)	
	milk	ND	50% (5/10)	0% (0/5)	ND	14.3% (1/7)	6.7% (1/15)	

		**Week 33**	**Week 35**	**Week 36**	**Week 37**			

**402**	blood	0% (0/22)	0% (0/35)	0% (0/27)	0% (0/19)			
	milk	0% (0/22)	12.5% (1/8)	0% (0/15)	0% (0/5)			

		**Week 15**	**Week 16**	**Week 17**				

**403**	blood	73.1% (19/26)	28.0% (7/25)	0% (0/26)				
	milk	100% (15/15)	18.5% (5/27)	4.3% (1/23)				

^a ^% reported is the proportion of virus quasispecies that did not have an amino acid substitution within the Gag p11C epitope (remained wild type).

^b ^In parentheses is the number of virus quasispecies that remained wild type at the Tat TL8 epitope/number of virus quasispecies sequenced.

^c ^ND = not done or unable to amplify virus variants

In summary, CTL escape of milk virus occurred with a pattern and rate similar to that seen in blood virus, suggesting that similar cellular immune pressures shape virus quasispecies evolution in these compartments. While SIV-specific CD8+ T lymphocyte responses during acute SIV infection appear in milk concurrently with the reduction of milk virus load from peak [15], we were not able to demonstrate that the high proportion of SIV-specific CD8+ T lymphocytes observed in milk drives earlier CTL escape of milk virus. However, the small number of evaluated monkeys and restricted sampling schedule make it difficult to demonstrate distinct rates of CTL escape in blood and milk virus. Although a limited number of animals were included in this study, clonal amplification of virus in milk and not in plasma of all animals studied suggests that replication of breast milk virus occurs locally.

## Competing interests

The authors declare that they have no competing interests.

## Authors' contributions

SP designed and coordinated the study, performed sequence data analysis, and drafted the manuscript. HK, AW, LM performed single genome amplification and sequencing. KG and AC participated in study design and supervised and performed nonhuman primate procedures. GL created the phylogenetic tree and performed phylogenetic sequence analysis. BH performed phylogenetic sequence analysis and assisted in data interpretation and manuscript writing. NL participated in the design and coordination of the study and assisted in the drafting of the manuscript.
